# Clients in Simulated Teletherapy via Videoconference Compensate for Altered Eye Contact When Evaluating Therapist Empathy

**DOI:** 10.3390/jcm11123461

**Published:** 2022-06-16

**Authors:** Frédéric Grondin, Anna M. Lomanowska, Vincent Poiré, Philip L. Jackson

**Affiliations:** 1 École de Psychologie, Université Laval, 2325 All. des Bibliothèques, Québec City, QC G1V 0A6, Canada; frederic.grondin.1@ulaval.ca (F.G.); vincent.poire.1@ulaval.ca (V.P.); 2 Center for Interdisciplinary Research in Rehabilitation and Social Integration (Cirris), 525 Wilfrid-Hamel Blvd, Québec City, QC G1M 2S8, Canada; 3 CERVO Brain Research Center, 2601 Chem. de la Canardière, Québec City, QC G1J 2G3, Canada; 4 Transitional Pain Service, Toronto General Hospital, University Health Network, 200 Elizabeth St., Toronto, ON M5G 2C4, Canada; anna.lomanowska@uhnresearch.ca

**Keywords:** eye contact, empathy, teletherapy, videoconferencing, eye tracking

## Abstract

Eye contact is frequently associated with an increased perception of empathy and telepresence, but the currently used videoconferencing (VC) technologies diminish the possibility of naturally conveying eye contact. This study compared the empathy, telepresence, and eye gaze patterns of clients in simulated VC teletherapy sessions where eye contact was altered or facilitated. Forty-two would-be clients met with one of four therapists in training for one 20-min simulated teletherapy session taking place via VC. The session either altered or facilitated eye contact perception by manipulating the positioning of the webcams and of the clients in their chair. Eye-tracking data focusing on the eyes, face, and general body regions of interest were obtained for 25 clients. The results show that facilitating eye contact in VC did not increase the clients’ perceptions of empathy or telepresence. However, empathy was associated with greater time spent looking at the eyes and faces of the therapists, but only in the sessions facilitating eye contact. We suggest that clients successfully rely on other verbal and nonverbal cues to detect therapist empathy when eye contact is altered in teletherapy sessions.

## 1. Introduction

Teletherapy refers to the use of technological means such as videoconferencing (VC) to allow for therapists and clients to meet for psychotherapy when they do not share the same physical space. Under the circumstances brought about by the COVID-19 global pandemic, teletherapy has become a widely-used alternative to in-person psychological treatments. A survey of the members of the American Psychological Association (APA) performed in June 2020 shows that 76% of clinicians who took part in the survey had completely transitioned to teletherapy at that time [1]. While it is currently uncertain how much of the recent uptake in teletherapy will persist beyond the global pandemic [2], there is a need to establish the effectiveness of teletherapy treatments and to identify areas where this clinical practice can be enhanced. A number of meta-analyses and systematic reviews support the claim that treatments delivered through VC are as effective as analog in-person interventions [3,4,5,6]. Establishing strong therapeutic alliance is also possible in teletherapy compared to in-person treatments according to numerous systematic reviews [7,8]. However, a recent meta-analysis found alliance to be weaker in teletherapy when integrating the results of 12 comparative studies between teletherapy and in-person therapy [5]. This last finding echoes the frequent concerns of therapists and clients that VC could impact the therapeutic relationship due to difficulties in perceiving and attending to nonverbal cues during VC sessions [9]. Technical limitations such as Internet connection instability, transmission delays, or equipment layout can lead to a relative loss of socioemotional cues called the “filtering effect” [10]. Nonverbal cues, more so than verbal content, thus run the risk of being altered or lost during VC-based interactions.

One such nonverbal behavior frequently compromised when interacting in VC is eye contact. Under typical circumstances, VC systems employed on home computers and laptops do not properly convey eye contact between the interactants due to the angle between the visual target on the screen (the other person’s eyes) and the webcam usually mounted atop the computer monitor. This gaze angle produces the impression for interlocutors that their counterpart is looking at their chin when intending to make eye contact. Although several adaptations have been proposed to facilitate eye contact in VC (for a review, see [11]), these adaptations are seldom used in clinical practice due to the need for advanced computational algorithms or specialized equipment. One possible solution that has the advantages of being simple and inexpensive for clinicians and clients is the thoughtful positioning of webcams and interlocutors to decrease the gaze angle to roughly 2° [12]. This solution, however, has not yet been implemented systematically and studied in teletherapy sessions.

The importance of facilitating eye contact in teletherapy sessions stems from its positive contribution to the subjective experience of clients during sessions, notably regarding empathy and telepresence. Empathy, by which therapists convey the emotional understanding and attunement integral to successful therapy [13], is rated higher in face-to-face counseling sessions where eye contact is encouraged. One study investigated the perceived empathy of observers within simulated sessions where the therapist either intended or avoided making eye contact [14]. Therapists were also instructed to modify their body posture to be either leaning forward or staying upright. The results showed that the “high eye contact” condition (intending to make eye contact most of the time) was associated with higher ratings of perceived empathy, regardless of body posture. These findings highlight the significance of eye contact for an observer when assessing the level of empathy of a therapist. Eye contact is also thought to play an important role in facilitating telepresence, or the impression of *being there* with the other person during a VC session [15]. One study found that participants who took part in a quiz with a confederate in a VC interaction reported higher telepresence in the quiz round where eye contact was enabled compared to the quiz round with altered eye contact [16]. Telepresence, incidentally, has also been suggested as a potential correlate of empathy, though the exact nature of the relationship is still debated [10]. Facilitating eye contact in teletherapy could, therefore, lead to the perception of both higher empathy and telepresence in VC.

Multiple methods can be employed to measure and quantify eye contact in human interactions [17]. The use of an eye-tracking apparatus offers the advantages of accurately and objectively measuring where and for how long a person is fixating their gaze. Examining gaze fixations toward specific regions of interests can provide valuable information for clinicians since gaze fixation can be indicative of prolonged attention toward specific visual information [18]. For example, the time a client spends fixating on different body parts (e.g., eyes, face, or body) of the therapist can be used as a marker of increased attention to visual cues expressed in these areas of the body (e.g., eye contact, facial expressions, or body movements). It is still unclear whether facilitating eye contact in VC therapy could result in different eye gaze patterns in clients for these regions of interests, for example, by increasing the time spent looking into the therapist’s eyes. A final advantage of precise eye tracking data is in the possibility of investigating correlations with self-reported measures such as the clients’ perception of empathy and telepresence.

This article presents the first empirical study to investigate the impact of facilitating eye contact in VC teletherapy on the empathy, telepresence, and eye-tracking patterns using simulated clinical sessions. The relationship between empathy, telepresence, and fixation times of the eyes, face, and general body was also investigated. The hypotheses were as follows:

**Hypothesis** **1** (**H1**)**.** *Empathy and telepresence reported by clients will be higher in VC teletherapy sessions where eye contact is facilitated*.

**Hypothesis** **2** (**H2**)**.** *Clients will spend more time looking into the eyes and the face of the therapist in VC teletherapy sessions where eye contact is facilitated compared to sessions where it is not*.

**Hypothesis** **3** (**H3**)**.** *Empathy and telepresence reported by clients will be correlated with the time spent looking into the face and the eyes of the therapists*.

**Hypothesis** **4** (**H4**)**.** *Empathy will be significantly correlated to telepresence in VC teletherapy*.

## 2. Materials and Methods

### 2.1. Participants

Forty-four students were recruited through posters and university mailing lists between October 2019 and March 2020, thus before the introduction of social distancing measures required by the advent of the COVID-19 pandemic. Participants were enrolled as clients if they were attending university courses at the time of the study and did not report uncorrected visual or auditory impairments. Clients reporting acute distress or suicidal thoughts received a list of resources, were offered to be accompanied through the process of contacting mental health services, and were excluded from the study. Clients were informed that they would be required to talk about a subject of personal nature of their choice with a therapist under training prior to completing the study questionnaires. The clients were randomly assigned to one of two experimental groups, More-EYE (with eye contact; *n* = 20) and Less-EYE (without eye contact, *n* = 23). One participant who completed the More-EYE condition was removed from the analyses because they misunderstood the experimental task, leaving a final total of 19 participants in the More-EYE group and an overall total of 42 participants. Table 1 presents the sociodemographic data of clients by experimental condition. Groups did not significantly differ based on their sociodemographic profile. Clients received CAD 15 for their participation in the study.

### 2.2. Materials

#### 2.2.1. Therapists

The therapists were four confederates enrolled in a doctoral degree in clinical psychology (aged 21, 24, 25, and 28 years old, three women). Each therapist had completed at least one year of supervised clinical practice. Therapists were informed that they would meet university students acting as clients in simulations of teletherapy sessions prior to the study but were blind regarding the objectives and hypotheses of the study. They each met 5, 9, 14, and 14 clients counterbalanced across the two groups.

#### 2.2.2. List of Discussion Themes

To encourage clients to choose a relevant topic of discussion while also ensuring that sensitive topics would not be discussed during the sessions, a list of potential themes of discussion used in helping skills training was provided to the clients (p. 19, [19]). Themes were classified as *ideal* (e.g., academic issues), *relatively safe* (e.g., minor family issues), or *proscribed topics* (e.g., traumas) for the context of the study. Frequently chosen topics included *Careers and future plans* and *Academic issues*.

#### 2.2.3. VC Sessions

The VC sessions were initiated using Zoom version 4.6.0 (Zoom Video Communications, Inc., San Jose, CA, USA) on cabled Internet and were displayed on 24-inch high-definition LCD computer monitors. Therapists and clients wore noise-cancelling headsets and sat in comfortable, adjustable chairs located in two separate lab rooms. This setup ensured that the quality of the video feed was as high as possible, with no noticeable video blurring or audio lag during the sessions. The VC sessions were recorded using OBS Studio, an open-source software for video recording. The two lab rooms were in the same building and close by, but the therapists and clients did not meet physically during the experimental task.

#### 2.2.4. Eye-Tracking Data

**Figure 1:**
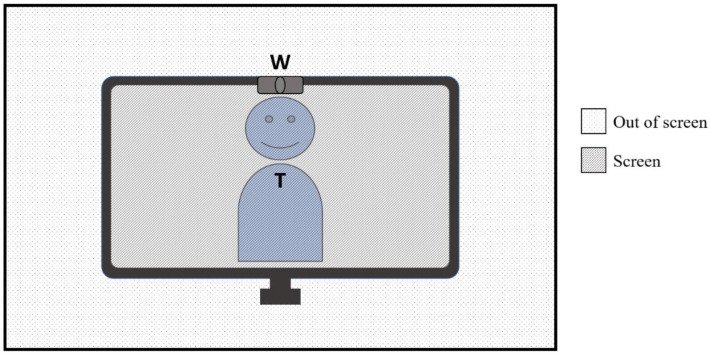
The depiction of *off-screen* versus *on-screen* portions of the visual scene from the client perspective. Legend: T: Therapist. W: Webcam.

**Figure 2:**
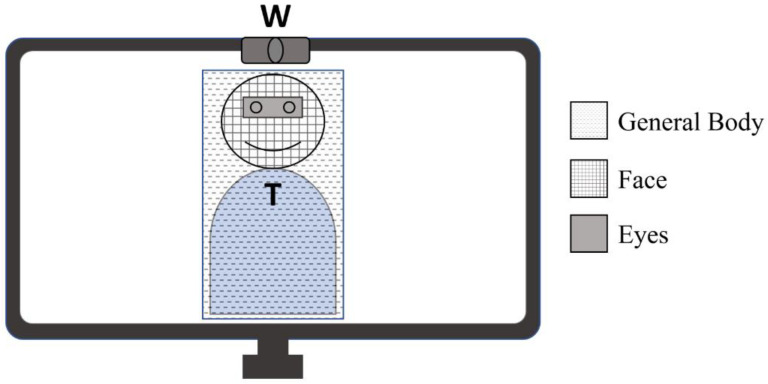
The delineation of the *eyes*, *face*, and *general body* regions of interest included in the *on-screen* portion of the visual scene from the client perspective. Legend: T: Therapist. W: Webcam.

The Smart Eye Pro 8 (Smart Eye Inc., Gothenburg, Sweden) eye-tracking system was employed to collect gaze coordinates and fixation information at a frequency of 120 hz. This system uses two synchronized monochromatic cameras (Basler acA640−120 gm) equipped with 6 mm lenses, infrared filters, and infrared flashes to register eye positioning and gaze direction. These cameras were placed 70 cm away from the clients, on each side of the desk on top of which the computer monitor was placed. To prepare the eye-tracking data and to compute the gaze ratios for each region of interest, scripts programmed in Python were used. The fixations identified by the eye tracking software were filtered by removing the fixations from the analysis where the duration was shorter than 70 ms or where the gaze signal quality was inferior to 10% using the thresholds suggested by Smart Eye [20]. The remaining fixations were classified as being directed *on-screen* or *off-screen* (Figure 1). The screen coordinate used for the *on-screen* fixations was the median value of the valid screen coordinates (x, y) provided by the eye tracker. The *on-screen* portion of the fixations were then classified into increasingly smaller and more specific areas of interest, beginning with a *general body* area, then a *face* area, and finally an *eyes* area (Figure 2). To extract the changing coordinates of the eyes and face across time, recordings of each session were first analyzed using a facial behavior analysis toolkit, OpenFace [21]. Body coordinates were manually entered for each recording to encompass the trunk of participants and added to the *face* and *eyes* coordinates to form the *general body* area. These parameters were then used to classify the data over time from the Smart Eye Pro system to extract fixation times relating to the three regions of interest. The resulting gaze duration for each area of interest was then compiled as a percentage ratio of the duration of *on-screen* gaze duration. Of the 42 total participants, 28 clients took part in the eye-tracking procedure and three of these 28 sessions were excluded from analysis following calibration failure or poor gaze detection quality. The final eye-tracking analyses comprised 13 clients for the More-EYE group and 12 clients for the Less-EYE group.

#### 2.2.5. Empathy

A French translation of the Empathic Understanding Subscale (EUS) of the Relationship Inventory was used [22]. The EUS is comprised of 16 items scored on a scale ranging between −3 to +3, with a positive score indicating higher perceived empathy. Upon inspecting the Cronbach alpha of the EUS scores, one item was consistently scored positively by clients when its intended scoring key indicated that the item should be reverse scored. This item (“The therapist understands what I say from an objective, detached point of view”) was therefore removed from the analyses. The total scores thus ranged from −45 to +45. The internal consistency analysis showed a Cronbach value of 0.85 for this study. The EUS is frequently used in studies investigating empathy in the context of therapy sessions [14,23].

#### 2.2.6. Telepresence

The French version of the Telepresence in Videoconference Scale (TVS) was used [15]. The scale includes seven items scored from 0 to 100 that are averaged into a total score. Three different subscales are also derived from the TVS: *Physical Presence,* defined as the impression of being in the same room as the therapist; *Social Presence,* defined as the impression of being in an ongoing social interaction with the therapist; and *Absorption,* defined as the impression of feeling immersed in the interaction. The TVS was especially developed to assess the clients’ perceptions of telepresence in teletherapy sessions. The TVS showed discriminant validity with associated constructs such as immersive tendencies and the participant’s comfort while using a communication medium such as VC [15]. The internal consistency analysis of the TVS revealed a Cronbach value of 0.80 for this study.

#### 2.2.7. Affectivity Changes

The French translation of the Positive Affectivity and Negative Affectivity Scale (PANAS) was used to assess the affectivity changes in clients following the session [24]. The PANAS includes 20 adjectives relating to the positive or negative effects that participants rate according to their level of agreement with each adjective on a 5-point Likert scale. The scores are aggregated on two subscales, *Positivity* and *Negativity*.

### 2.3. Procedure

At the start of the study, therapists took part in an hour-long training course aimed at clarifying their role in the study. Therapists were instructed to explore the discussion theme chosen by the clients with warmth, interest, and openness as they normally would in their clinical practice. They were, however, instructed to refrain from conducting actual therapeutical interventions as the sessions were aimed at simulating teletherapy only. They concluded the training course by completing one 20-min session in VC that served to validate the methodology used for facilitating eye contact in VC (see [12]).

**Figure 3:**
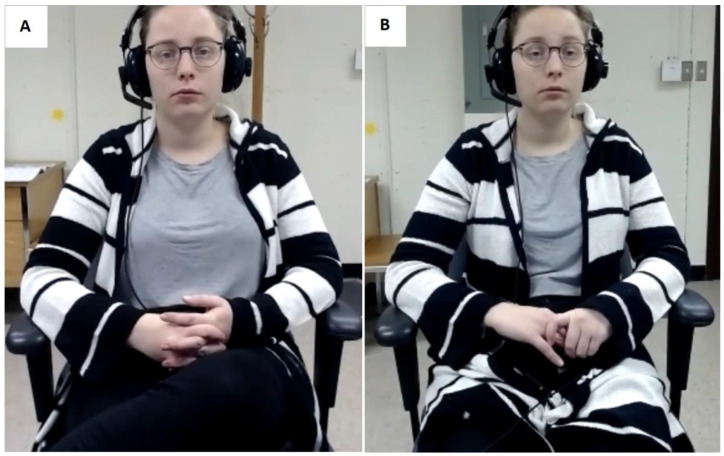
The clients’ view of the therapist under the two experimental conditions: (**A**) the More-EYE condition with facilitated eye-contact, and (**B**) the Less-EYE condition with altered eye-contact.

Clients who took part in the study were greeted in a lab room and were reminded that their participation consisted of speaking with a therapist in training about a subject of a personal nature of their choice. Clients such as therapists were not informed that the study examined eye contact, empathy, and telepresence in the VC sessions prior to their completion of the task. Prior to the session, participants completed the consent form, the sociodemographic questionnaire, and the pre-session PANAS. Next, the eye-tracking calibration procedure of the Smart Eye Pro 8 was performed in the 28 sessions where eye-tracking data were registered. Clients then met with the therapists in VC under one of two experimental conditions, the More-EYE and the Less-EYE condition. The More-EYE condition facilitated eye contact perception by diminishing the gaze angle down to approximatively 2°. The More-EYE condition was set up by following a procedure that was developed to minimize the gaze angle by adjusting the positioning of the webcam and of the interactants [12]. This layout ensures that participants looked almost directly into the webcams, which enabled the perception of eye contact in VC. The Less-EYE condition introduced a 10° horizontal gaze angle to ensure that the eye contact perception was altered during the sessions (see Figure 3). Therapists, unlike clients, were always under the More-EYE condition, which was thought to be the ‘ideal’ experimental condition.

Each VC session lasted 20 min. The experimenters were invited into the Zoom call as an invisible guest to allow for the recording of the session as it unfolded using OBS Studio. Participants received a signal at the 19-min mark to leave some time for the termination of the session. After the session, clients completed the empathy, the telepresence, and the post-session affectivity questionnaires. Participants were then debriefed and received monetary compensation.

### 2.4. Analyses

SPSS 22.0.0.0 (IBM Corp., Armonk, NY, USA) was used to perform the statistical tests and the alpha level was set at 0.05. Statistical corrections for multiple comparisons or correlations were not applied to the data as the analyses were hypothesis-driven and the number of tests per analysis was low. Effect sizes of *t*-tests were generated using Cohen’s *d* formula [25].

## 3. Results

To test whether perceived empathy and total telepresence, respectively, increased when eye contact was facilitated in the VC sessions (H1), two one-tailed *t*-tests for independent samples were performed on the empathy and telepresence mean scores (Table 2). Perceived empathy and telepresence did not differ significantly between the More-EYE and Less-EYE conditions. The level of empathy (e.g., [23]) and telepresence (e.g., [26]) reported by clients was high for both conditions in comparison to earlier studies using the same questionnaires. Two-tailed *t*-tests were also performed to compare each of the three subscales of the telepresence questionnaire across conditions and no significant differences were found (Table 2). These results indicate that clients did not report more empathy or telepresence in the sessions where eye contact was facilitated.

To test whether the clients’ gaze distribution differed between the More-EYE and Less-EYE conditions (H2), a Mann–Whitney *U* test was used to compare the *on-screen* time ratios between the More-EYE and the Less-EYE condition (Table 3). These analyses included eye tracking data from the 25 sessions where the Smart Eye Pro 8 was available. The Mann–Whitney U test was selected over parametric tests to account for the non-normal distribution of the eye-tracking data. Regions (*eyes*, *face*, *general body*) were not compared between themselves as they were comprised within one another (see Figure 2). This first test showed no significant differences in the time ratios across conditions, indicating that participants spent a similar amount of time looking at the computer screen across conditions. Three Mann–Whitney *U* tests were then performed to compare the mean fixation times for the *eye*, *face*, and *general body* regions (Table 3). The mean fixation durations for the *eye* and the *face* regions were not significantly different across conditions. The mean *general body* fixation duration was significantly higher in the More-EYE condition, indicating that participants tended to look more at the therapists in the More-EYE condition than in the Less-EYE condition.

To test whether perceived empathy and telepresence were associated with time spent looking at the *eyes*, *face*, or *general body* of therapists across conditions (H3), the Spearman Rho correlations (*r_s_*) were calculated for each of the two experimental conditions (Table 4). The Spearman Rho correlations were selected based on the non-normality of the eye-tracking data. In the More-EYE condition, empathy marginally correlated (*p* = 0.06) with the time spent looking at the *eyes* of the therapist and significantly correlated with the time spent looking at the *face*. In the Less-EYE condition, these correlations were not statistically significant. These results indicate that the time clients spent looking at the eyes of the therapist was associated with their perceived level of therapist empathy, but only in the More-EYE condition. In both experimental conditions, telepresence did not significantly correlate with the mean gaze duration in any of the three regions of interest.

To test whether empathy ratings were correlated to the telepresence *total scores* as well the *physical presence*, *social presence*, and *absorption* subscales (H4), Pearson correlations were computed in the More-EYE and Less-EYE conditions (Table 5). The results indicate that empathy correlated significantly with the *social presence* subscale in the Less-EYE condition only.

Manipulations checks were performed using the affectivity changes reported on the affectivity questionnaire. This was conducted to verify whether clients benefitted from the session in the way that they presumably would in an actual teletherapy session. Four two-tailed *t*-tests for paired samples were performed using pre- and post-session levels of positivity and negativity for each of the two conditions. Three clients in the Less-EYE condition encountered technical difficulties with their pre-session affectivity computerized form, bringing the total observation count to 20 for these analyses. The results showed a significant decrease in negativity in the More-EYE condition and a marginally significant (*p* = 0.052) increase in positivity in the Less-EYE condition (Table 6). These results indicate that clients benefitted from the sessions but in different ways between the experimental conditions.

Table 7 features the *eyes* and *face* region sizes in pixels for each eye contact condition as well as the *eyes-to-face* ratios between these regions. Mann–Whitney analyses for paired samples were performed to test for differences in the size and ratio between the eye contact conditions. This was carried out to verify whether the manipulation of the camera angle between conditions had an effect on the size of the therapist’s *face* and *eyes* regions on the screen that clients looked at during sessions. The Mann–Whitney analyses showed that the *eyes* region was marginally bigger and that the *face* region was significantly bigger in the More-EYE condition compared to the Less-EYE condition. The *eyes-to-face* ratios did not differ significantly. These results indicate that the manipulation of the camera angle resulted in bigger *eyes* and *face* areas for the More-EYE sessions compared to the Less-EYE sessions.

To explore how eye contact unfolded over time, the time spent looking at the therapist’s eyes was compared across 5 min segments of the 20 min interaction in the More-EYE and the Less-EYE conditions combined. Table 8 shows the mean and median time spent looking at the eyes of the therapist for each 5 min segment. A Friedman’s test (the nonparametric equivalent of a repeated-measures ANOVA) was performed on the time spent looking at the therapist’s eyes for all clients across the 5 min segments. The Friedman’s test showed no overall significant change in time spent looking at the therapist’s eyes, χ^2^(3) = 5.044, *p* = 0.17.

## 4. Discussion

### 4.1. Hypothesis 1: Empathy and Telepresence Ratings across Sessions

This study aimed at determining whether facilitating eye contact in VC sessions would lead to heightened impressions of perceived empathy and telepresence compared to sessions where eye contact was altered. Two experimental conditions were compared in this study: one facilitating eye contact (More-EYE) and one altering eye contact (Less-EYE). The results showed that both experimental conditions produced high ratings of empathy and telepresence compared to previous studies (e.g., [14,23]), with no significant influence of eye contact facilitation on reports of empathy and telepresence. This finding is contrary to the initial hypotheses. It appears that solely manipulating the perception of eye contact in VC does not greatly impact the perception of empathy and telepresence. There are multiple possible explanations for the lack of significant differences between the groups. First, although participants could not naturally establish eye contact in the Less-EYE condition, earlier research showed that VC users rapidly learned to interpret the deviated gaze of their interlocutor as intended eye contact [27]. It is therefore possible that this knowledge was sufficient for clients to assess that their therapist was attentive to their subjective experience, a perception that would be conducive to a high rating of empathy regardless of the possibility to actually establish eye contact or not. Another explanation for the lack of significant differences relates to the overall favorable parameters of the sessions. Participants had a reliable, high-quality video feed to support their interaction in both conditions, potentially allowing clients to perceive empathy, even when eye contact is altered. These explanations would need to be tested in contexts where video and audio feeds fluctuate in quality and where the view of the therapist is restricted to assess the impact of these factors on perceived empathy. In terms of telepresence, the results obtained from the *Absorption* subscale showed a high level of variance in both conditions. This suggests that the clients’ impression of being absorbed in the interaction varied according to factors outside of the availability of eye contact during sessions, thus affecting the statistical analysis of this variable across experimental conditions.

### 4.2. Hypothesis 2: Clients’ Time Spent Looking at Regions of Interest across Sessions

The study also investigated whether facilitating eye contact influenced the time spent looking at the eyes, face, and general body of therapists compared to sessions where eye contact was not facilitated. The results showed that clients spent more time looking at the body of therapists in the More-EYE condition but, contrary to the hypotheses, they did not significantly look more at the eyes or the face of the therapists than the participants in the Less-EYE condition. In contrast, the data pointed to a nonsignificant increase (*p* = 0.16) in time spent looking at the eyes of the therapist in the Less-EYE condition rather than in the More-EYE condition. One way to reconcile these conflicting results is by reconsidering the assumption that facilitating eye contact would lead clients to look *more* in the eyes of the therapists rather than *less*. In an in-person interaction, prolonged eye contact can cause discomfort, and this effect could be compounded by the experimental task where clients had to open up on a subject of a personal nature to a stranger [28]. However, when eye contact is altered, as it was in the Less-EYE condition, it is possible for clients to fixate more on the eyes of the therapists without feeling discomfort. Therefore, it seems possible that clients in the More-EYE condition tried to avoid eye contact once they had the indication that the therapist was paying attention to them and instead focused on looking elsewhere on the screen, thus leading to the significant increase in time spent looking at the general body of the therapists. It is interesting to note that the exploratory analyses of the time clients spent looking at the therapist’s eyes showed no significant change over the course of the 20-min session (Table 8). In future studies, it would be pertinent to examine whether the time spent looking at the therapist’s eyes changes over a longer session or across multiple sessions of teletherapy.

### 4.3. Hypothesis 3: Correlations between Time Spent Looking at Regions of Interest and Empathy and Telepresence across Sessions

The study aimed at identifying whether empathy and telepresence were associated with time spent looking at the eyes, face, and general body of therapists with the hypothesis that more time would be spent looking at the eyes and face in the More-EYE condition. The results showed a marginally significant and a significant correlation between time spent, respectively, looking at the eyes and face of the therapists in the More-EYE condition. These correlations were not observed in the Less-EYE condition. These results lend support to the contention that eye contact is an important indicator of empathy from the perspective of clients, as the clients who experienced facilitated eye contact in VC tended to rate empathy higher as they spent more time making eye contact. However, when considering that no significant difference in empathy was found between the conditions, it seems plausible that clients do not necessarily need proper eye contact to perceive high therapist empathy and that, in the presence of altered eye contact, they adapt by basing their assessment of therapist empathy on other available cues. For example, body posture [14], facial expressions [29], vocal cues [30], and verbal interventions [31] can also influence perceived empathy. These cues were all preserved in the sessions regardless of the experimental condition and thus could have helped clients make a favorable judgement of therapist empathy when eye contact was not facilitated.

### 4.4. Hypothesis 4: Correlations between Empathy and Telepresence

The results showed that empathy was not significantly correlated with the total telepresence scores in either experimental condition. The investigation of the telepresence subscales revealed that empathy correlated with the social presence subscale in the Less-EYE condition only. Broadly speaking, these results do not support the contention that empathy and telepresence are strongly correlated in VC sessions. However, there seems to be a specific aspect of telepresence related to the impression of being actively participating in an ongoing online interaction with someone (i.e., the ratings on the *social presence* subscale) that is more highly rated in sessions where the clients also report high empathy. The fact that this correlation was found only in the Less-EYE condition also lends more credence to the hypothesis outlined above regarding the possibility that clients rely on factors other than eye contact to rate therapist empathy when eye contact is not facilitated. In other words, when eye contact is altered, clients who feel like they are part of an ongoing interaction with the therapist are likely to also report high empathy. This result, however, needs to be expanded upon with further research to properly identify the aspects of a VC clinical session that are conducive to feeling more *present* in an online interaction.

### 4.5. Limitations

Some limitations need to be considered along with the findings of this study. The use of a simulated clinical session is useful to bridge the gap between the state of research surrounding empathy in teletherapy and clinical practice, but it is possible that clients undergoing actual treatment in teletherapy would experience the sessions differently than the clients in the experimental sessions. Still, the participants in this study did discuss a personal theme with a therapist in training and they reported benefits in terms of affectivity, lending support to the assumption that the experimental task was a faithful simulation of actual teletherapy.

Another limitation that pertains to the eye-tracking measurements comes from the compromises that were made to manipulate the gaze angle while also making it possible to record the eye-tracking data during the sessions. For instance, participants sat 70 cm away from the eye-tracker, which is in the upper limit of the tolerated distance between the eye-tracker cameras and the participant according to the lenses employed. The computer monitor itself stood 50 cm behind the eye-tracker, thus 1.2 m away from the participants. The uncertainty of measurement of the eye-tracker is typically under 1°, which translates to having an uncertainty of approximatively 2 cm on any given eye-tracking measurement. This uncertainty of measurement could have had a systematic impact on the recorded eye-tracking data, particularly on the eyes’ region of interest where the eye-tracking signal could have been miscategorized as being directed toward the face region rather than the eye region. The authors still considered that the validity of the results was sufficient to provide meaningful insights regarding the eye-tracking patterns of the participants since both experimental conditions were impacted in the same way by the uncertainty of measurement. This systematic error could potentially be circumvented with an even more precise eye-tracking setup in future studies. Additionally, the results showing that the *eyes* and *face* regions were significantly bigger in the More-EYE condition (see Table 7) implies that the time spent looking at the eyes and face of the therapist in this experimental condition could have been amplified compared to the Less-EYE condition by the increased size of the *eyes* and *face* regions (see Figure 2 for an illustration of the size of each region). However, these results do not impact the interpretation of other analyses since the *eyes* and *face* regions, albeit bigger in size, were not looked at longer in the More-EYE condition than in the Less-EYE condition (see Table 3). Moreover, the aims of this study regarding the correlations involving the time spent looking at the *eyes* and *face* regions and empathy did not involve comparing the More-EYE correlations to the Less-EYE correlations. The systematic increase in the region size in the More-EYE condition is therefore unlikely to affect the correlational results found in Table 4.

Finally, it should be noted that the participants and therapists in this study were mostly female. Previous studies have shown that female participants are more likely to feel and convey higher levels of empathy than male participants [32]. This sex effect might therefore have inflated the empathy levels in both conditions, though the sex ratios were similar between groups (see Table 1). The therapists also met a similar number of clients in each eye contact condition. Future studies should investigate whether the sex of clients and therapists could influence the levels of empathy found in teletherapy settings.

## 5. Conclusions

Though eye contact seems to be an important indicator of empathy, this study suggests that participants playing the role of clients were able to perceive a high degree of empathy in a typical VC session where eye contact is altered by the positional offset of the webcam compared to the eyes of the therapist on the screen. Future studies are needed to identify other factors that may contribute to increased perceived empathy in teletherapy as they could be a target of interventions to optimize the clients’ experience of teletherapy. These factors include camera framing [33] and the quality of the video feed. Investigating the role of nonverbal behaviors in actual teletherapy sessions could also yield important information on the mechanisms underlying the perception of empathy in teletherapy.

## Figures and Tables

**Table 1:** The sociodemographic data of clients across the experimental conditions.

Variables	More-EYE Condition (*n* = 19)	Less-EYE Condition (*n* = 23)	*p* Value ^a^
	Mean (SD)	Mean (SD)	
Age (years)	23.21 (3.84)	24.22 (5.38)	0.50
Sex (female)	15	20	0.49
Years of education	16.42 (2.09)	17.26 (3.41)	0.36
Chronic medication (Yes) ^b^	0	3	0.10
History of mental health disorders (Yes) ^c^	2	6	0.20
History of clinical consultation as a client (Yes) ^d^	11	9	0.35
Computer Use			
Days/week	6.58 (0.84)	6.87 (0.34)	0.28
Hours/day	5.63 (2.63)	6.22 (2.21)	0.43
Videoconferencing use			
Days/week	0.74 (0.93)	1.44 (1.90)	0.36
Hours/week	0.71 (0.99)	1.50 (1.81)	0.19

^a^ The reported *p* values were obtained from independent *t*-tests for the *Age* and *Years of education* variables. Mann–Whitney *U* tests were performed on the *Computer Use* and *Videoconferencing Use* variables because of their non normal data distribution. Chi-squared tests were performed for the *Sex*, *Chronic Medication*, *History of mental health disorders*, and *History of clinical consultation as a client* variable. These tests used an alpha level of 0.05 for significance. ^b^ Chronic medication included the use of psychotropic medication only such as antidepressants and painkillers. ^c^ History of mental health disorders comprised mood disorders, anxiety disorders, or eating disorders, but excluded attention deficit disorder with or without hyperactivity. ^d^ History of clinical consultation in psychotherapy as a client.

**Table 2:** The comparisons of empathy and telepresence across the experimental conditions.

Variables		More-EYE		Less-EYE	*t*	*p*	Cohen’s *d*
	*n*	Mean (SD)	*n*	Mean (SD)			
Empathy ^a^	19	28.11 (9.31)	23	24.91 (12.63)	0.915	0.18	0.28
Telepresence ^b^—total	19	71.28 (14.11)	23	69.13 (10.84)	0.558	0.29	0.17
Telepresence—physical	19	77.63 (19.68)	23	76.30 (15.18)	0.247	0.81	0.08
Telepresence—social	19	89.08 (8.79)	23	85.76 (14.39)	0.878	0.39	0.20
Telepresence—absorption	19	60.79 (21.73)	23	64.57 (26.13)	−0.502	0.62	0.16

^a^ Empathy = Empathic Understanding Subscale. ^b^ Telepresence = Telepresence in Videoconference Scale.

**Table 3:** The comparisons of the fixation durations of the percentage ratios across conditions.

Fixation Duration (%)	*n*	More-EYE	*n*	Less-EYE	*U*	*p*
		Mean (SD)		Mean (SD)		
On-Screen ^a^	13	77.20 (14.80)	12	74.97 (8.68)	62.00	0.41
Eye ^b^	13	7.37 (11.20)	12	13.99 (19.37)	51.50	0.16
Face ^b^	13	69.91 (26.95)	12	66.63 (29.69)	67.00	0.57
General body ^b^	13	90.68 (6.49)	12	80.85 (19.90)	38.00	0.03 *

^a^ Ratio out of total session duration. ^b^ Ratio out of on-screen gaze duration. * *p* < 0.05.

**Table 4:** The correlations between empathy, telepresence, and time spent looking at the eyes, face, and general body of the therapists.

Fixation Ratios ^a^		Empathy ^b^	Telepresence ^c^
**More-EYE (*n* = 13)**			
Eyes	*r_s_*	0.53	−0.37
	*p*	0.06	0.21
Face	*r_s_*	0.57	−0.29
	*p*	0.04 *	0.34
General body	*r_s_*	−0.19	−0.11
	*p*	0.54	0.71
**Less-EYE (*n* = 12)**			
Eyes	*r_s_*	−0.17	0.33
	*p*	0.60	0.29
Face	*r_s_*	−0.22	0.40
	*p*	0.49	0.20
General body	*r_s_*	0.10	0.21
	*p*	0.77	0.52

^a^ Ratios out of on-screen gaze duration. ^b^ Empathy: Empathic Understanding Subscale. ^c^ Telepresence: Telepresence in Videoconference Scale. * *p* < 0.05.

**Table 5:** The correlations between empathy and telepresence.

	*r* with Empathy ^a^	*p*
**More-EYE (*n* = 19)**		
Telepresence ^b^—total	−0.21	0.39
Telepresence—physical	0.09	0.71
Telepresence—social	0.24	0.33
Telepresence—absorption	−0.18	0.46
**Less-EYE (*n* = 23)**		
Telepresence—total	0.31	0.15
Telepresence—physical	0.35	0.10
Telepresence—social	0.81	<0.001 *
Telepresence—absorption	−0.11	0.62

^a^ Empathy: Empathic Understanding Subscale. ^b^ Telepresence: Telepresence in Videoconference Scale. * *p* < 0.05.

**Table 6:** The client affectivity ^a^ scores pre- and post-session.

	*n*	Pre-Session	Post-Session	*t*	*p*	Cohen’s *d*
		Mean (SD)	Mean (SD)			
**More-EYE**						
Positivity	19	32.25 (4.24)	33.95 (3.87)	1.624	0.12	0.35
Negativity	19	15.50 (4.29)	12.60 (2.98)	−3.368	0.003 *	0.67
**Less-EYE**						
Positivity	20	30.20 (5.60)	32.90 (5.48)	2.077	0.052	0.46
Negativity	20	14.15 (4.18)	13.10 (4.00)	−1.437	0.167	0.32

^a^ Affectivity: Positive Affectivity and Negative Affectivity Scale. * *p* < 0.05.

**Table 7:** The *eyes* and *face* region sizes and *eyes-to-face* ratios ^a^ between the eye contact conditions.

	*n*	More-EYE	*n*	Less-EYE	*U*	*p*
		Mean (SD)		Mean (SD)		
*Eyes* region size ^b^	13	16,989.69 (5066.59)	12	13,907.35 (2394.85)	43.00	0.060
*Face* region size	13	29,236.52 (5066.59)	12	24,367.60 (3398.98)	41.00	0.046 *
*Eyes-to-face* ratio	13	0.580 (0.052)	12	0.573 (0.061)	68.00	0.611

^a^ Ratio between the *eyes* region on the total size of the *face* region. See Figure 2 for each region delineation on the screen. ^b^ Region size in pixels. * *p* < 0.05.

**Table 8:** The time spent looking at the therapist’s eyes per 5 min segment of the interaction for all clients.

Segments (min)	*n*	Time Spent Looking at the Eyes of the Therapist (%)	
		Mean (SD)	Median
0–5	25	5.34 (7.26)	1.29
5–10	25	5.98 (10.40)	0.63
10–15	25	8.22 (12.54)	2.72
15–20	25	7.74 (11.07)	2.22

## Data Availability

A request has been made to make available all data reported in this study through an institutional repository. A reference number will be provided as soon as it is available.

## References

[1] American Psychological Association Psychologists Embrace Telehealth to Prevent the Spread of COVID-19 2020. http://www.apaservices.org/practice/legal/technology/psychologists-embrace-telehealth.

[2] Aafjes-van Doorn K., Békés V., Prout T.A. (2020). Grappling with our therapeutic relationship and professional self-doubt during COVID-19: Will we use video therapy again?. Couns. Psychol. Q..

[3] Berryhill M.B., Culmer N., Williams N., Halli-Tierney A., Betancourt A., Roberts H., King M. (2019). Videoconferencing Psychotherapy and Depression: A Systematic Review. Telemed. e-Health.

[4] Fernandez E., Woldgabreal Y., Day A., Pham T., Gleich B., Aboujaoude E. (2021). Live psychotherapy by video versus in-person: A meta-analysis of efficacy and its relationship to types and targets of treatment. Clin. Psychol. Psychother..

[5] Norwood C., Moghaddam N.G., Malins S., Sabin-Farrell R. (2018). Working alliance and outcome effectiveness in videoconferencing psychotherapy: A systematic review and noninferiority meta-analysis. Clin. Psychol. Psychother..

[6] Turgoose D., Ashwick R., Murphy D. (2018). Systematic review of lessons learned from delivering tele-therapy to veterans with post-traumatic stress disorder. J. Telemed. Telecare.

[7] Simpson S.G., Reid C.L. (2014). Therapeutic alliance in videoconferencing psychotherapy: A review. Aust. J. Rural Health.

[8] Watts S., Marchand A., Bouchard S., Bombardier M. (2016). L’alliance thérapeutique lors d’une télépsychothérapie par vidéoconférence pour un trouble du spectre anxieux: Recension systématique des écrits. Rev. Québécoise Psychol..

[9] Connolly S.L., Miller C.J., Lindsay J.A., Bauer M.S. (2020). A systematic review of providers’ attitudes toward telemental health via videoconferencing. Clin. Psychol. Sci. Pract..

[10] Grondin F., Lomanowska A.M., Jackson P.L. (2019). Empathy in computer-mediated interactions: A conceptual framework for research and clinical practice. Clin. Psychol. Sci. Pract..

[11] Regenbrecht H., Langlotz T. (2015). Mutual gaze support in videoconferencing reviewed. Commun. Assoc. Inf. Syst..

[12] Grondin F., Lomanowska A.M., Békés V., Jackson P.L. (2020). A methodology to improve eye contact in telepsychotherapy via videoconferencing with considerations for psychological distance. Couns. Psychol. Q..

[13] Elliott R., Bohart A.C., Watson J.C., Murphy D. (2018). Therapist Empathy and Client Outcome: An Updated Meta-Analysis. Psychotherapy.

[14] Dowell N.M., Berman J.S. (2013). Therapist nonverbal behavior and perceptions of empathy, alliance, and treatment credibility. J. Psychother. Integr..

[15] Berthiaume M., Bouchard S., Brisebois C., Robillard G. The Validation of a Telepresence Scale for Psychotherapy Delivered in Videoconference. Proceedings of the CYPSY23 23rd Annual CyberPsychology, CyberTherapy & Social Networking Conference.

[16] Neureiter K., Moser C., Tscheligi M. (2014). Look into My Eyes & See, What You Mean to Me. Social Presence as Source for Social Capital. Int. Conf. Soc. Inform..

[17] Jongerius C., Hessels R.S., Romijn J.A., Smets E.M.A., Hillen M.A. (2020). The Measurement of Eye Contact in Human Interactions: A Scoping Review. J. Nonverbal. Behav..

[18] Gwizdka J., Dillon A., Fu W.T., van Oostendrop H. (2020). Eye-Tracking as a Method for Enhancing Research on Information Search. Understanding Search Information and Improving: A Cognitive Approach.

[19] Hill C.E. (2014). Helping Skills: Facilitating Exploration, Insight, and Action.

[20] (2019). Smart Eye Pro Tech Support. Personal communication.

[21] Schroff F., Kalenichenko D., Philbin J. FaceNet: A unified embedding for face recognition and clustering. Proceedings of the IEEE Conference on Computer Vision and Pattern Recognition.

[22] Barrett-Lennard G.T. (1962). Dimensions of therapist response as causal factors in therapeutic change. Psychol. Monogr. Gen. Appl..

[23] Marci C.D., Ham J., Moran E., Orr S.P. (2007). Physiologic Correlates of Perceived Therapist Empathy and Social-Emotional Process during Psychotherapy. J. Nerv. Ment. Dis..

[24] Gaudreau P., Sanchez X., Blondin J.P. (2006). Positive and negative affective states in a performance-related setting testing the factorial structure of the PANAS across two samples of French-Canadian participants. Eur. J. Psychol. Assess..

[25] Cohen J. (1988). Statistical Power Analysis for the Behavioral Sciences.

[26] Germain V., Marchand G., Bouchard S., Drouin M.-S., Guay S. (2009). Effectiveness of Cognitive Behavioural Therapy Administered by Videoconference for Posttraumatic Stress Disorder. Cogn. Behav. Ther..

[27] Grayson D.M., Monk A.F. (2003). Are You Looking at Me? Eye Contact and Desktop Video Conferencing. ACM Trans. Comput.-Hum. Interact..

[28] Sharpley C.F., Sagris A. (1995). Does eye contact increase counsellor-client rapport?. Couns. Psychol. Q..

[29] Sharpley C.F., Jeffrey A.M., Mcmah T., Sharpley C.F., Jeffrey A.M., Mcmah T. (2007). Counsellor facial expression and client-perceived rapport. Couns. Psychol. Q..

[30] Imel Z.E., Barco J.S., Brown H.J., Baucom B.R., Kircher J.C., Baer J.S., Kircher J.C., Atkins D.C. (2014). The Association of Therapist Empathy and Synchrony in Vocally Encoded Arousal. J. Couns. Psychol..

[31] Wynn R., Wynn M. (2006). Empathy as an interactionally achieved phenomenon in psychotherapy: Characteristics of some conversational resources. J. Pragmat..

[32] Christov-Moore L., Simpson E.A., Coudé G., Grigaityte K., Iacoboni M., Ferrari P.F. (2014). Empathy: Gender effects in brain and behavior. Neurosci. Biobehav. Rev..

[33] Nguyen D.T., Canny J. (2009). More than face-to-face: Empathy effects of video framing. Proceedings of the SIGCHI Conference on Human Factors in Computing Systems.

